# Zebrafish as a Neuroblastoma Model: Progress Made, Promise for the Future

**DOI:** 10.3390/cells10030580

**Published:** 2021-03-06

**Authors:** Shuai Li, Kok Siong Yeo, Taylor M. Levee, Cassie J. Howe, Zuag Paj Her, Shizhen Zhu

**Affiliations:** 1 Department of Biochemistry and Molecular Biology, Mayo Clinic College of Medicine, Rochester, MN 55902, USA; sli@lcmail.lcsc.edu (S.L.); yeo.koksiong@mayo.edu (K.S.Y.); Levee.Taylor@mayo.edu (T.M.L.); howe.cassie@mayo.edu (C.J.H.); Her.ZuagPaj@mayo.edu (Z.P.H.); 2 Department of Molecular Pharmacology & Experimental Therapeutics, Center for Individualized Medicine, Mayo Clinic College of Medicine, Rochester, MN 55902, USA

**Keywords:** neuroblastoma, zebrafish, animal model

## Abstract

For nearly a decade, researchers in the field of pediatric oncology have been using zebrafish as a model for understanding the contributions of genetic alternations to the pathogenesis of neuroblastoma (NB), and exploring the molecular and cellular mechanisms that underlie neuroblastoma initiation and metastasis. In this review, we will enumerate and illustrate the key advantages of using the zebrafish model in NB research, which allows researchers to: monitor tumor development in real-time; robustly manipulate gene expression (either transiently or stably); rapidly evaluate the cooperative interactions of multiple genetic alterations to disease pathogenesis; and provide a highly efficient and low-cost methodology to screen for effective pharmaceutical interventions (both alone and in combination with one another). This review will then list some of the common challenges of using the zebrafish model and provide strategies for overcoming these difficulties. We have also included visual diagram and figures to illustrate the workflow of cancer model development in zebrafish and provide a summary comparison of commonly used animal models in cancer research, as well as key findings of cooperative contributions between MYCN and diverse singling pathways in NB pathogenesis.

## 1. Introduction

Over the past ten years, zebrafish have become an increasingly popular tool for scientists conducting biomedical studies and other research. The species’ high fecundity rate, low cost of maintenance, and the ease of observation and genetic manipulation all contribute to its increasing use as an alternative and valuable vertebrate model system to study human disease. The expanding community of researchers using zebrafish has brought advanced technologies to the model, as well as a rapidly expanding inventory of transgenic and mutant lines that can be applied to different research niches. Cancer research using the zebrafish model can be traced back to 1965, when Dr. Mearle Stantion performed pioneered work to induce hepatic neoplasia in zebrafish with Diethylnitrosamine [1]. In 2003, the first zebrafish genetic cancer model was reported by Drs. David Langenau and Thomas Look, in which the MYC oncogene was overexpressed under control of the *rag2* promoter, resulting in the development of T cell leukemia in the transgenic animal [2]. Since then many more zebrafish cancer models have been developed to understand the pathogenesis of leukemia, melanoma, rhabdomyosarcoma, hepatocellular carcinoma and many other tumor types [3,4,5,6]. In particular, the zebrafish model has also shown exceptional promise in dissecting the contributions of genetic alterations that were identified from integrative genomic analyses of neuroblastoma (NB) to the pathogenesis of this devastating pediatric cancer.

NB is the most common extracranial solid tumor in children and accounts for ~10% of all childhood cancer-related deaths [7]. It is derived from transformed neural crest progenitor cells in the developing peripheral sympathetic nervous system (PSNS) [8,9]. High-risk patients with amplified *MYCN* and over 18 months of age are often presented with widespread metastasis at diagnosis. Over the past few years, the five-year event-free survival rate for children with high-risk disease remains lower than 50% [10,11]. Very recently, a Phase III trial of immunotherapy, consisting of Dinutuximab, granulocyte macrophage-colony stimulating factor (GM-CSF) and interleukin-2 (IL2), showed significantly increased five-year overall survival rate of patients with high-risk NB to ~70% [12,13]. This immunotherapy has been approved by FDA for the treatment of patients with high-risk NB who achieve at least a partial response to prior first-line multiagent, multimodality therapy [12]. Although the improved outcomes are observed with the inclusion of Dinutuximab as part of treatment regimens for newly diagnosed NB, the prognosis for the relapsed disease remains poor (<10% progression-free survival) [14,15]. Therefore, better understanding of the pathogenesis of this disease and developing novel and more effective therapies are needed.

As an important member of the *MYC* proto-oncogene family identified from NB patients [16], *MYCN* amplification accounts for ~25% of NB cases and is associated with poor disease outcome [17,18,19]. *MYCN* is a bHLH transcription factor and is homologous to c-MYC structurally and functionally. It can promote neoplastic transformation of cultured mammalian cells and rat embryo fibroblasts [20,21]. In 1997, Dr. William Weiss developed the first animal model of NB by overexpressing *MYCN* under control of Tyrosine Hydroxylase (*TH*) in transgenic mice, which is by far still the most popular model for NB research [22]. Following Dr. Weiss’s effort, several genetically modified mouse (GEMM) lines with direct, conditional, inducible overexpression, knock-in or knockout of NB-relevant genes, including mutationally activated *ALK* (Anaplastic Lymphoma Receptor Tyrosine Kinase) [23,24,25], *LIN28B* (Lin-28 Homolog B) [26,27], *SV40* large T antigen (Simian Vacuolating Virus 40 TAg) [28,29,30] and others [31,32] were subsequently developed. These models demonstrated a sufficient induction of NB in mice, which resemble the features of human NBs [24].

Although the mouse model provides valuable molecular insights on NB pathogenesis and opened the door for NB research, it has some disadvantages when compared to the zebrafish model. Neuroblastomas are different from adult tumors, in that they arise early in development; identifying the early onset of tumorigenesis in mice without euthanizing the animals is difficult and creates challenges in dissecting the molecular and cellular mechanisms underlying early onset tumor initiation. Zebrafish, by contrast, are translucent and develop from externally fertilized eggs, which allows for early detection of tumor onset in live animals. The zebrafish model is also more practical than GEMM, less expensive, and does not require sacrificing these animals to track tumor initiation and visualization of tumor growth. Therefore, the zebrafish model can serve as an alternative for the commonly used mouse model to conduct genetic research.

**Figure 1:**
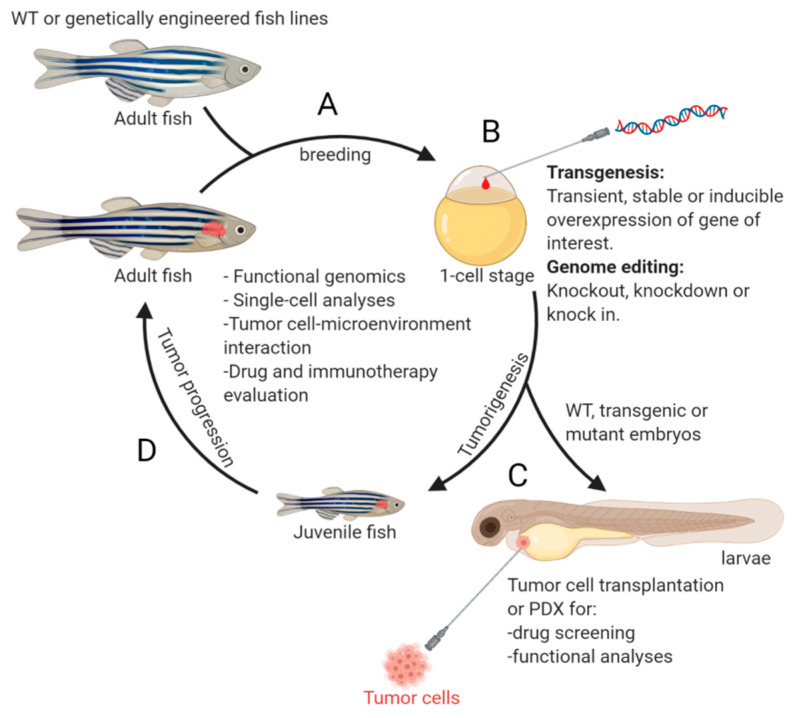
An overview of the workflow using zebrafish model for NB study. Offspring from mating of wild-type (WT) or genetically engineered fish lines (**A**) can be subjected for (i) genome editing or transgene overexpression at one-cell stage (**B**), or (ii) transplantation of tumor cells at 2 days post fertilization for subsequent drug screening or functional analyses (**C**). The genetically modified embryos (**B**) can also be raised up for monitoring tumor development (**D**). Examples of crucial studies that can be performed using the zebrafish model are listed in the middle of the circle. This figure was created with BioRender.com.

In 2012, the first zebrafish model of NB was generated and published by Zhu et al. [33]. Two oncogenes, *MYCN* and mutationally activated *ALK* (the most commonly mutated genes in primary neuroblastoma [34,35,36,37] and an attractive candidate for targeted therapy [38,39]), were expressed under control of the dopamine-beta-hydroxylase (*dβh*) promoter [33]. Following this initial effort on modeling NB in zebrafish, many new transgenic fish lines were developed, uncovering additional novel genetic alterations that cooperate with MYCN or c-MYC during NB pathogenesis. The evolution of zebrafish NB models has revealed the complexity of this disease at the molecular level and demonstrates the robustness of the model system in deepening our understanding of the molecular and cellular basis underlying NB pathogenesis. An overview of the NB zebrafish disease model workflow is illustrated in Figure 1.

**Figure 2:**
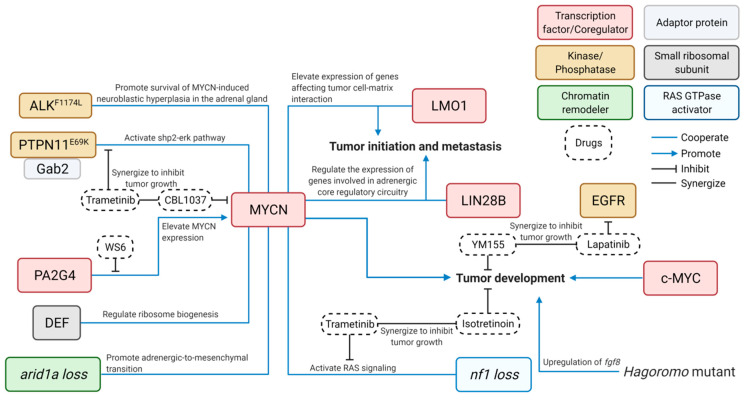
Cooperative contributions of diverse signaling pathways to the pathogenesis of NB—findings from zebrafish models. Blue lines connect cooperative genes in NB pathogenesis; Blue arrows indicate positive impact; Bar-headed lines indicate inhibitory effect; and Black lines indicate synergy between drugs. *ALK*, anaplastic lymphoma kinase; *arid1a*, AT-rich interacting domain–containing protein 1A; *c-MYC*, V-Myc avian myelocytomatosis viral oncogene homolog; *DEF*, digestive organ expansion factor; *EGFR*, epidermal growth factor receptor; *Gab2*, GRB2-associated-binding protein 2; *LIN28B*, lin-28 homolog B; *LMO1*, LIM domain only 1; *MYCN*, V-Myc avian myelocytomatosis viral oncogene neuroblastoma; *nf1*, neurofibromatosis type 1; *PAG2G4*, proliferation-associated protein 2G4; and *PTPN11*, protein tyrosine phosphatase non-receptor type 11. This figure was created with BioRender.com.

Of course, the advances in understanding NB have not been achieved without obstacles and challenges, some of which appear daunting. In this paper, we aim to:

1. Compare and contrast the zebrafish model with other popular lab animals used as disease models in order to help cancer and other biomedical researchers determine appropriate models for experimental applications (Table 1); and
2. Provide an objective overview of the advancements in research that have occurred using the zebrafish NB model (Figure 2 and Table 2).

Since topics related to PSNS development in zebrafish and mammals and NB genetics have been previously covered in detail by ourselves and others [40,41], we will not be addressing these subjects in this review.

## 2. Advantages of Using Zebrafish as a Model for NB Research

### 2.1. Translucency of Juvenile and Adult Fish

The translucent appearance of both embryonic and adult zebrafish makes this species an obvious choice for NB study, since they can be observed while unharmed with the naked eye or under a fluorescent microscope using fluorescent probes (Table 1).

#### 2.1.1. Early Detection of Tumor Onset

For the *TH-MYCN* mouse model, palpation is still the most common method used by researchers to examine tumor formation [22,55]. However, because tumors predominantly originate from the abdominal ganglion structures [22], it can be technically challenging for researchers to detect the early onset of tumorigenesis and track the rate of tumor progression by touch. Recently, PET or MRI scans have been used to evaluate the growth of *TH-MYCN* tumors in the presence or absence of different compounds, such as the Aurora A kinase or mTOR inhibitor [59,60]. These techniques seem promising and sensitive for monitoring tumor progression. However, they require expensive equipment, trained professionals, and lengthy procedures. In contrast, the onset of NB can be easily detected by fluorescent microscopy in the zebrafish model and large cohorts of animals (over 400 fish) can be monitored efficiently and regularly without harming the fish as early as 5 weeks post fertilization (wpf) [33,47,48]. Therefore, the zebrafish model allows demonstration of the cooperative contributions of multiple genetic alterations to NB tumorigenesis with high statistical power.

#### 2.1.2. Real-Time Monitoring of Tumor Progression and Metastasis

In addition to being useful in the early detection of tumor onset, fluorescent-tagged tumor cells can be monitored for the growth of tumor, especially tumor metastasis in real-time. In transgenic fish with overexpression of *MYCN* and *LMO1* oncogenes, fluorescent-positive tumor masses were observed using fluorescent microscopy in the distant regions from the primary tumor site, the interrenal gland region (IRG), as early as five weeks of age [48]. Using pathological and immunohistochemical analyses with antibodies against neuroblastoma markers, such as Tyrosine Hydroxylase (TH) [61] and HuC [62], widespread metastasis were detected in the orbit, gill, spleen, distal portion of kidney and heart [48]. Additional sites of metastases, such as the bone, the liver and the pancreas, were also observed in another newly developed zebrafish model with loss of function of *gas7* gene [58].

Although there are anatomical differences between zebrafish and mammals, most of the organs in mammals have their functional equivalent counterparts in zebrafish. For example, the zebrafish IRG is equivalent to the human adrenal gland, which contains the chromaffin cells (an important cell lineage of origin for NB [63]) interposed with interrenal epithelial cells in the head kidney [64]. Similarly, although the lymph nodes are absent in zebrafish, the spleen and kidney (the sites where T cells, B cells, and dendritic cells reside) serve as secondary lymphoid organs in zebrafish [65,66]. The kidney marrows also function similarly to mammalian bone marrow [65]. The gills of zebrafish have a similar structure to the mammalian airways and fulfill the same gas-exchange function, making it analogous to the mammalian lung [67,68]. With such functional, physiological and anatomical homology of zebrafish to mammals, the metastases detected in the fish model match quite well to those metastatic sites commonly seen in human NB patients, including the bone marrow (70%), bone (55%), lymph nodes (30%), liver (30%), and brain (18%) [69]. In addition, increased numbers and networks of picrosirius red-stained collagen fibers, indicating the enhanced stiffness of extracellular matrix, were observed in the fish tumors with overexpression of both *MYCN* and *LMO1* [48], which is consistent with the concept that increased ECM stiffness contributes to enhanced metastasis [70,71]. Hence, the zebrafish model offers a unique advantage in dissecting the molecular and cellular basis and the contribution of microenvironment to NB metastasis.

#### 2.1.3. Efficient Evaluation of the Efficacy of Drug Treatment

It is challenging to develop effective and safe targeted therapies for cancer patients, especially for children. To do so requires using an entire organism to test the efficacy of new drugs or novel combinations thereof and evaluate the drug toxicity in order to develop new targeted therapies. Several NB-bearing zebrafish models have been used to demonstrate the effectiveness of novel inhibitors and combinations of new compounds in the treatment of NB, along with illuminating of the underlying mechanisms that may contribute to drug resistance in NB therapy (Figure 2).

For instance, using the *dβh-MYCN* zebrafish NB model (designated the *MYCN* line), Radic-Sarikas et al. demonstrated that the epidermal growth factor receptor (EGFR) kinase inhibitor, lapatinib, can prolong and enhance the cytotoxicity of YM155, an anti-cancer drug, by inhibiting the multidrug-resistance efflux transporter ABCB1 [49]. This led to the synergistic inhibition of the growth of *MYCN*-overexpressing NB in vivo. Similarly, using the transgenic fish with overexpression of both *MYCN* and proliferation associated 2G4 (*PA2G4*), Koach et al. demonstrated that a small molecule, WS6, can competitively bind to PA2G4 to prevent its interaction with MYCN, leading to destabilization and reduced expression of MYCN and in turn suppressed growth of NB [55]. Using the same zebrafish *MYCN* model together with the transgenic fish overexpressing both *MYCN* and *GAB2*, Zhang et al. demonstrated that the MEK inhibitor trametinib can enhance the sensitivity of *MYCN* and *GAB2*-overexpressing NB to the treatment of CBL0137, a histone chaperone FACT inhibitor [47]. In addition, He et al. applied the MEK inhibitor (trametinib) and retinoid (isotretinoin) on the juvenile compound fish with loss of *nf1* in the context of *MYCN* overexpression and demonstrated synergistic killing of tumor cells by this combination treatment [46]. Taken together, these zebrafish NB models showed considerable translational potential for investigating new strategies to improve the treatment of this devastating childhood tumor.

### 2.2. Robustness in Genome Editing and Manipulation of Gene Expression

Since fish deposit oocytes outside of the body for external fertilization, researchers can easily collect fertilized embryos in large batches to perform genetic editing at the single-cell stage effectively and efficiently. The whole process—from breeding the fish to injections of transgene constructs or genome editing reagents into a few hundred embryos—takes only two days. Zebrafish are relatively simple to use when compared to rodent models that require multiple time-consuming steps including a period of superovulation by pregnant mare serum (PMS) in the early afternoon followed by human chorionic gonadotropin treatment [72], labor-intensive oocyte harvesting, in vitro fertilization, and embryo implantation.

The relative ease-of-use of zebrafish embryos compared to mice allows researchers to rapidly and economically develop a large number of genetically engineered fish lines in a short period of time. Various genetic editing methods have proven effective in zebrafish and this article will focus on approaches used in NB research and how they may be applicable and transferable to other research settings.

#### 2.2.1. Retroviral-Mediated Mutagenesis

Retrovirus-mediated insertional mutagenesis in zebrafish was established in 1996 [73]. It is a powerful forward-genetic approach for identifying genes that are critical for embryonic development [74].

In 2009, led by Drs. Nancy Hopkins and Jacqueline Lees, a team of researchers performed a comprehensive insertional mutagenesis screen in zebrafish with a goal to identify genes susceptible to cancer. Four mutant fish lines with viral insertions in the fbxw4 gene were identified. Interestingly, the mis-regulated fibroblast growth factor 8 (*fgf8*) in these mutants was found to contribute to neuroblastomagenesis [42].

Although the forward-genetic approach was successful, it was quite challenging to screen for fish with spontaneous development of NB and to effectively identify disease-driving genes. Therefore, the reverse-genetic strategy became a more popular approach to model NB using the zebrafish in the past decade.

#### 2.2.2. I-SceI Meganuclease-Mediated Transgenesis

Using a reverse-genetic approach, the first transgenic zebrafish model of neuroblastoma was developed in Dr. Thomas Look’s laboratory in 2012. Two stable transgenic lines with overexpression of *MYCN* or *ALK*^F1174L^ were generated using meganuclease (I-SceI)-mediated transgenic strategy [33]. It is important to note that human genes, rather than zebrafish genes, are commonly chosen for developing a transgenic zebrafish NB model. Doing so allows researchers to avoid the potential issues of targeting transgenes or validating the efficacy of drugs designed to interact with human proteins.

The I-SceI-mediated transgenesis is highly efficient in zebrafish and frogs, although the transgene integration rate seems low in other species [75,76]. The strategy to build transgene constructs for a NB zebrafish model is similar to that of the *TH-MYCN* mouse model. However, the *TH* promoter did not work well in zebrafish. Therefore, the *dβh* promoter was used instead to drive expression of genes of interest in the PSNS [33]. The *dβh-EGFP-MYCN* construct was used to generate the first zebrafish NB model (designated as MYCN line). Due to the instability of MYCN protein, EGFP-MYCN expression was visible under fluorescence microscopy but was not strong [33]. Hence, researchers applied a strategy of co-injection of *dβh-EGFP* or *dβh-mCherry* DNA with *dβh* promoter-driven transgene constructs containing genes of interest for the subsequent development of multiple transgenic lines.

The co-injected DNA has been demonstrated to be co-integrated into the fish genome, leading to their co-expression in the PSNS cells [33]. The EGFP expression is detectable in the embryos as early as one-day post fertilization [33]. Hence, the EGFP-positive embryos can be easily identified and sorted using fluorescent microscopy, which eliminates additional genotyping procedures after the transgenic line is confirmed. This approach is cost-efficient for conducting large-scale experiments (which require screening hundreds of transgenic fish) at a time when lab space is at a premium.

Using a similar transgenesis approach, researchers have developed a panel of transgenic lines with overexpression of control reporters or genes of interest. These include transgenic fish lines overexpressing *EGFP* [33] or *mCherry* [48] alone as a control group, or NB-relevant genes including *ALKwt* [33], *PTPN11* [47], *GAB2* [47], *LIN28B WT* [56], *LIN28B_MU* [56], *LMO1* [48], and *DEF* [50]. Interestingly, most of the aforementioned genes have been shown to cooperate with MYCN to contribute to the pathogenesis of NB through different cellular mechanisms (Figure 2). However, none of these genes can sufficiently drive NB tumorigenesis when they were overexpressed alone, suggesting that MYCN is a potent oncogene and a key driver for the NB initiation.

Recently, a newer stable transgenic zebrafish line with overexpression of *MYCN* under control of the *dβh* promoter was developed in Dr. Thomas Look’s lab [50]. Instead of using the DNA construct carrying a *dβh-EGFP-MYCN* fusion gene, two transgenic constructs, *dβh-MYCN* (cDNA+3′UTR) and *dβh-EGFP*, were coinjected into the one-cell stage of wild-type embryos in developing this line (designated as *TgMYCN_TT*). The advantage of the coinjection approach has been illustrated above. The major difference of the *TgMYCN_TT* line to the *MYCN* line is the inclusion of the 3′ UTR of *MYCN* gene, containing microRNA recognition sites [77], in the transgene construct. Thus, the *MYCN* expression in the *TgMYCN_TT* line could be regulated at the post-transcriptional level, which might link to the increased penetrance of NB to ~70% by 29 wpf [50] in the *TgMYCN_TT* line as compared to ~30% in the *MYCN* line by 25 wpf [48]. Moreover, overexpression of *c-MYC*—a highly expressed MYC-family gene in the *MYCN* non-amplified high-risk NBs—in the PSNS of transgenic fish could also induce NB tumorigenesis [51], thus implicating the key oncogenic role of MYC family genes in NB pathogenesis.

#### 2.2.3. Genome Editing with Clustered Regularly Interspaced Short Palindromic Repeats (CRISPR) and Transcription Activator-Like Effector Nucleases (TALENs)

CRISPR can effectively target genes of interest and achieve up to 75–99% modification rate in zebrafish [78,79]. This high-throughput targeting can generate close to 30% germline mutations [80] with as low as 1% of off-target effects [81]. Shi et al. generated *arid1aa* and *arid1ab* knockout zebrafish using this robust technology in the NB model with *MYCN* overexpression to examine the relevance of *ARID1A* as a NB suppressor gene in vivo and found that both *arid1aa* and *arid1ab* deficiency increases penetration of *MYCN*-driven NB in zebrafish [57]. Alternatively, targeted deletion in the zebrafish genome can be achieved efficiently using the TALENs system [82]. With this method, a *gas7* knockout fish line was generated and bred with the *MYCN* transgenic line, which led to development of widespread metastasis in the compound fish [58].

#### 2.2.4. Other Potentially Useful Methods

Given that zebrafish modeling for NB research has only become common in the last decade, there are still a plethora of research methods in the zebrafish that have not been applied to the NB study, but that have potential to be used in the near future. For example, the Cre-Lox recombination, a site-specific recombinase technology, has been widely used in different model systems. Briefly, a ubiquitously or tissue-specifically expressed tyrosine recombinase enzyme (Cre) can be used to recombine a pair of short target sequences, called the Lox sequences, leading to manipulation of DNA sequence at specific sites. Such Cre-LoxP conditional expression approach has been used to target the oncogenic Kras/Ras pathway under control of the *nestin* promoter [83]. Given that many lineage-specific promoters are available in zebrafish, this system is highly applicable when studies require specific activation of gene of interest in a given subset of cell population. For example, Langenau et al. was able to express mouse c-Myc in lymphoblasts of zebrafish using this experimental approach [84]. Heat shock inducible promoters also appear to be applicable which has been used to track individual retina-neuron axon pathways [85]. Additionally, tamoxifen-inducible Cre recombinase can conditionally activate gene expression when needed, such as CreERT2 in zebrafish [86]. The whole system can be easily integrated using CRISPR/Cas9 in zebrafish [87]; a detailed review on the zebrafish Cre-LoxP system has previously been published [88].

### 2.3. High Throughput Transplantation, Patient-Derived Xenograft (PDX) and In Vivo Drug Screening Using Zebrafish Larvae

To understand disease pathogenesis and screen or validate drug efficacy in vivo, scientists have successfully transplanted tumor cells with different genetic alteration(s) or manipulated gene expression, as well as patient-derived tumor cells into zebrafish at embryonic stage or adulthood [89]. Several features of zebrafish larvae make them uniquely suited for these studies, including: (i) transparent bodies that allow for easy tumor cell injection; (ii) ability to use trackable fluorescent-tagged cells following transplantation; (iii) an immature immune system during early embryonic development, which reduces the chance of the immune rejection of transplanted tumor cells; and (iv) availability of large clutches of embryos for transplantation. Multiple injection sites, such as the perivitelline space, pericardial space, yolk, retro-optical region, and brain, have been explored in a variety of studies to understand the mechanisms of tumor metastasis, angiogenesis, cellular intravasation/extravasation [90,91,92].

Drug screening on zebrafish transplants or xenografts is another exemplary usage of this model. Both embryos and adults can be used in high-throughput drug-screening assays. Embryos are relatively easy to work with due to their permeability of small molecules [93]. Researchers have already performed small-molecule drug screening using zebrafish embryos transplanted with neural crest stem cells (NCSCs) [54]. Since NB is derived from the sympathoadrenal lineage of neural crest cells, small molecules that inhibit NCSC induction might be potentially useful for the NB treatment. Among the 640 FDA-approved drugs applied in this screen, one drug, leflunomide, was identified to inhibit NCSC induction. Leflunomide, as an inhibitor of dihydroorotate dehydrogenase (DHODH) and an immunosuppressive agent for the treatment of patients with rheumatoid arthritis, has already been shown to reduce proliferation and induce apoptosis in NB cells both in vitro and in vivo [94]. Hence, this result further demonstrates the important application of zebrafish as an unbiased in vivo system for effective drug screening. Recently, zebrafish transplanted with human NB cells have been used to demonstrate the effect of a new multi-kinase drug, TP-0903, on reducing extravasation and inducing tumor cell death, suggesting the therapeutic potential of this compound for the NB treatment [53].

Although PDX mouse models are considered the gold standard for the in vivo validation of drug efficacy, the studies led by Drs. Ferreira and Fior, have demonstrated that the patient-derived zebrafish xenografts (zPDX, also called cancer “avatars”) can be used to sense cancer behavior and screen for potential novel therapies. Using a panel of zebrafish xenografts with patient-derived colorectal cancers, Ferreira and Fior rapidly screened the available therapeutic options for the colorectal cancers and predicted the treatment outcomes [92,95], which set the groundwork for using zPDX as a rapid in vivo screening platform for future personalized cancer treatments. Following these efforts, a high-throughput zebrafish xenograft assay of neuroblastoma was performed to confirm cannabinoid receptor 2 (CNR2) and Mitogen-activated protein kinase 8 (MAPK8) as promising candidates for the treatment of high-risk NB and to identify the drugs GW405833 and AS601245 as the most effective and well-tolerated CNR2 and MAPK8 targeted compounds to inhibit the growth of xenografts in zebrafish [96].

To better mimic the cytokine-enriched microenvironment found in human patients for xenotransplantation, Dr. Berman’s group generated the first humanized zebrafish by overexpressing transgenes encoding human hematopoietic-specific cytokines, such as GM-CSF, stem cell factor (SCF), or stromal cell-derived factor 1α (SDF1α). Transgenic lines with overexpression of each of the individual gene mentioned above were developed first using Tol2 transposon-mediated transgenic approach and then incrossed to generate a compound transgenic fish line with overexpression of all of the aforementioned cytokines (GM-CSF, SCF, and SDF1α) (designated GSS fish) [97]. Patient-derived leukemias transplanted into the GSS zebrafish exhibit improved survival, self-renewal ability and broader clonal representation. Therefore, the GSS fish establish a new standard for zebrafish xenotransplantation that more accurately recapitulates the human context for evaluating personalized treatment [97].

## 3. Potential Challenges of Zebrafish as a Model for Cancer Research

The zebrafish model for studying NB is not without its drawbacks and challenges. However, as we will demonstrate in this section, most challenges are not unique to researching neuroblastomas, but rather are general issues that arise from conducting in vivo research using zebrafish.

### 3.1. Temperature for Husbandry

Zebrafish are cold-blooded vertebrates that typically live in an aquatic environment with a temperature range between 28 and 30 degrees centigrade—significantly lower than the body temperature of approximately 37 degree centigrade for humans and mice. Therefore, in vivo experiments, such as transplantation of human NB cells and NB patient-driven xenografts, may not be optimal [98]. However, zebrafish can tolerate environments that are on par or exceed the temperature of normal human physiology, as laboratory zebrafish have demonstrated that a critical thermal maxima (CTmax) of up to 41 degrees centigrade is endurable [99]. Therefore, fish acclimation through incubation at a higher temperature can be achieved after transplantation or xenograft experiments. These specialized conditions require dedicated infrastructure and monitoring, which may be a challenge in some laboratories.

It has been shown that the zebrafish embryos transplanted with human cells can be directly maintained at 36°C without significantly impacting the viability of fish for 72 h [100]. The injected cells in the embryos incubated at 36 °C showed higher proliferation rate than those incubated at 34°C [100]. Besides the zebrafish embryos, PDX and human cell lines can also be transplanted into adult immune compromised zebrafish and incubated for a longer period of time at human body temperature. Zebrafish can tolerate human body temperate after a week of acclimation. A gradual temperature increase between 1–2 °C per day and the presence of antibiotics are needed. After developing serval critical immunocompromised fish lines, the Langenau lab successfully engrafted 16 different types of human cancer cells and PDXs in the *prkdc-/-*;*il2rga-/-* adult mutant fish which can be reared at 37°C [101]. Their data further demonstrated the feasibility of maintaining zebrafish at a human physiological temperature and the xenograft experiments in zebrafish model can recapitulate similar results obtained in the mouse model [101].

### 3.2. General Physiological Differences and Conserved PSNS Development in Zebrafish

As non-mammalian vertebrates, zebrafish are physiologically distinct from humans and mice in multiple and significant ways, including the lack of anatomical analogues to human lungs, breasts, prostates, etc. Thus, a zebrafish model may not be suitable for research to model tumors that commonly develop in the aforementioned organs. However, the development of sympathoadrenal lineage of the neural crest, from which neuroblastoma cells are derived, is highly conserved between zebrafish and mammals [9,102,103,104], making zebrafish a reliable model for NB studies.

Further evidence of the reliability of the zebrafish model has been provided by research showing that overexpression of *MYCN* in the zebrafish PSNS under control of the *dβh* promoter induces adrenal sympathetic neuroblast hyperplasia [33], which is consistent with the observation of hyperplastic lesions in the adrenal glands of LSL-*MYCN*;Dbh-iCre mice [105] or in sympathetic ganglia of *TH-MYCN* mice [22,106], the other two popular NB mouse models with *MYCN* overexpression. Further characterization of the tumors that arose from the *MYCN*-overexpressing transgenic fish by histopathological, immunohistochemical and ultrastructural analyses demonstrated their similar features to human NB [33]. In addition, the hematogenous metastases of tumor cells to the similar common sites of metastases seen in patients were also observed in the transgenic fish lines with overexpression of *MYCN* and *LMO1* or *LIN28B* [48,56].

### 3.3. Gene and Genome Variation

The zebrafish genome was first fully sequenced in 2013 [107]. The most current version of gene annotation for this species was released in 2018 via the Ensembl genome database. It has been reported that 71.4% of human genes have their orthologues in zebrafish [107]. Due to the teleost-specific genome duplication, zebrafish have more than 26,000 protein coding genes [108]. In comparison, there are approximately 21,000 protein-coding genes in human DNA [109]. Within the human pool of protein-coding genes, 9528 have an equivalent zebrafish orthologue, 3105 human genes have multiple orthologues in the zebrafish genome [107]. Accumulating evidence from comparative analyses have revealed that an apparent genome-wide duplication occurred in the ancestry of zebrafish [110,111]. Such genome duplication leads to more than one variant of zebrafish homologues to human homologues, which affects ~15% of human coding genes and likely results in gain of novel gene functions or unique expression patterns [112]. For example, several tumor-relevant genes, such as *pten* [113], *nf1* [114], *ptpn11* [115] or *arid1a* [57], are duplicated in zebrafish and their variants have both overlapping and non-redundant functions in embryonic development and/or tumorigenesis, including in NB tumorigenesis. Hence, understanding the roles of duplicated genes—especially genes that suppress tumors—requires knockout of both homologs from the zebrafish genome, which is a potentially time-consuming and a technically challenging endeavor.

## 4. Future Applications of the Zebrafish for NB Research

The rapid development of novel technologies in zebrafish and cancer research provides new opportunities and challenges for application of the zebrafish model in NB studies. Since the first landmark work of using the single-cell approach to reconstruct developmental trajectories and mapping gene expression landscapes as well as lineages during zebrafish embryogenesis in 2018 [116,117], the single-cell RNA sequencing (scRNAseq) strategy has been quickly adapted to study various developmental processes [118,119,120] and pathogenesis of different types of diseases in zebrafish models [121]. As it has been shown earlier in the zebrafish NB model, overexpression of *LMO1* upregulates the expression of genes affecting tumor cell-extracellular matrix interaction and promotes NB cell invasion and migration [48]. The advances in single-cell technologies, including the opportunities to delineate the heterogeneous states of tumor cells and their interactions with non-tumor cells or microenvironment, would help to further uncover the mechanisms of neuroblastoma progression and metastasis. Moreover, the response of tumor cells to drug treatments can be evaluated at the single-cell level in zebrafish, which could provide scientists with a better understanding of the molecular basis that underlies drug resistance, the major obstacle in effective NB treatment.

Over the last decade, immunotherapy has significantly improved survival rates in many patients with hematologic malignancies and adult solid tumors. NB has been a positive example of immunotherapy in pediatric solid tumors [13,122]. Although immunotherapeutic approaches have shown promising results for pediatric solid tumors in early clinical trials [122], significant clinical benefit for NB has not be realized. Therefore, the zebrafish could serve as a tool to optimize immunotherapy-based approaches to improve disease outcomes. One of the special challenges of immunotherapy in many types of pediatric solid tumors is the relatively high percentage of cold tumors with paucity of T-cell infiltration into the immunosuppressive tumor microenvironment [122]. Hence, the approaches to promote recruitment of T-cells to the tumors to convert phenotypically “cold” into “inflamed” tumors might potentially improve the therapeutic effect. Very recently, zebrafish have been used for an in vivo visualization of the anti-tumor activity of tumor-infiltrating lymphocytes (TILs) and chimeric antigen receptor T (CAR-T) cells to co-transplanted human primary melanoma cells [123]. Thus, this study provides a valuable in vivo platform for future validating the efficacy of T cell-mediated immunotherapy and screening for effective antibodies or agents that could revert tumor microenvironments from immunosuppressive to immunoactive to improve immunotherapy in childhood cancers, including NB.

## 5. Conclusions

Even though the zebrafish model is relatively recent, it has already emerged as a valuable model and powerful tool for researchers seeking to unlock the molecular and cellular mechanisms underlying the pathogenesis of this cancer and working to develop effective treatments. Efforts by us and others over the past decade on developing zebrafish models for the study of neuroblastoma has provided an increased understanding of this devastating childhood cancer. Future applications for this model could potentially include the studies of genetics, epigenetics, tumor microenvironments, oncological immunotherapies, single-cell transcriptomics, precision medicine, etc.

In concert with the power of real-time in vivo imaging, effective transplantation of human xenografts, high-throughput screenings of therapeutic agents alone or in combination, and robust tracking cell–cell or cell-microenvironment interactions at single-cell level, this unique model opens a myriad of paths towards a greater understanding, better treatments, and hopefully one day a cure for this devastating childhood cancer.

## Figures and Tables

**Table 1:** Comparison of commonly used lab animal models in cancer research.

	Zebrafish	Mouse	Fly	Worm
Transparency	Fully transparent at embryonic stage and remain translucency through adulthood. PTU can be used to inhibit pigmentation during early embryonic development. Mutant fish line without pigments are available.	Not transparent	Transparent in larva stage and some parts of the adults	Transparent No pigmentation
Offspring size per mating	Up to 100	~3–12	Up to 500	Hermaphrodites, varies
Genetic similarity (humans genome as reference)	71%	85%	50%	52%
Immune System	Underdeveloped adaptive immune system in larvae	Intact	Does not possess acquired/adaptive immunity	Does not possess acquired/adaptive immunity
Tumor visualization	Directly visualized in vivo by microscopy	Cannot be easily visualized inside the body	Directly visualized in vivo by microscopy	Directly visualized in vivo by microscopy
Gene editing tools				
Morpholino	Established	Feasible but very limited	Possible but not done yet	Possible but not done yet
Retroviral insertion mutagenesis screen	Feasible	Established	Feasible	Feasible
DNA co-injection (I-SceI) Transgenesis	Established, high efficiency	Hypothetical and not efficient	Hypothetical	Possible
CRISPR/TALENs	Established	Established	Established	Established
Tumor transplantation/Xenograft application	Efficient	Moderate to difficult	N/A	N/A
Chimeric animal development	Mouse-zebrafish Chimeric	Human-mouse Chimeric	N/A	N/A
Syngeneic model	Yes	Yes	Yes	N/A
Drug screening	Established, high-throughput	Established, low-throughput	Established, high-throughput	Established, high-throughput

**Table 2:** Zebrafish models for the studies of the PSNS development and NB pathogenesis as well as evaluation of the efficacy of anti-NB drugs.

Publications	Approaches	New Models Developed	Drugs Tested in the Zebrafish Models	Drugs applied in NB Treatment, Clinical Trials or other Animal Models
Amsterdam, A. et al., 2009 [42]	Retroviral-mediated mutagenesis	*Hagoromo* Mutants	N/A	N/A
Zhu, S. et al., 2012 [33]	I-SceI meganuclease mediated transgenesis	*Tg(dβh:EGFP-MYCN*) and *Tg(dβh:EGFP; dβh:ALK^F1174L^*) transgenic fish lines	N/A	N/A
Pei, D. et al., 2013 [43]	Morpholino-mediated gene knockdown & transient overexpression of structure variants	Embryos with gain or loss of function of *phox2b*/*PHOX2B*	13–cis retinoic acid (at 1~100 nM) treatment of embryos	Applied to patients with high-risk NB as maintenance therapy after consolidation therapy [44,45]
He, S. et al., 2016 [46]	I-SceI meganuclease mediated transgenesis	*Tg(dβh: GRD; dβh:mCherry*) transgenic fish line	Isotretinoin (13-cis retinoic acid, at 1~2 µM) and Trametinib (MEK inhibitor, at 10~40 nM) treatment of juvenile fish	Trametinib is in clinical trials for the treatments of various types of cancers, including high-risk NB (see NCI clinical trial information).
Zhang, X. et al., 2017 [47]	I-SceI meganuclease mediated transgenesis	*Tg*(*dβh:Gab2wt*;*dβh:EGFP*) and *Tg*(*dβh:ptpn11^E69K^*-*EGFP*) transgenic fish lines	CBL0137 (FACT inhibitor, at 4 mM) and Trametinib (MEK inhibitor, at 2 μM) treatment of tumor-bearing fish	CBL0137 is in a clinical trial for the treatment of patients with advanced extremity melanoma or sarcoma with metastasis (see NCI clinical trial information). In TH-MYCN tumor-bearing mice, CBL0137 combined with panobinostat can ablate tumor completely (Oncology Times: December 20, 2018)
Zhu, S. et al., 2017 [48]	I-SceI meganuclease mediated transgenesis	*Tg*(*dβh:LMO1*;*dβh:mCherry*) transgenic fish line	N/A	N/A
Radic-Sarikas, B. et al., 2017 [49]	Drug treatment	N/A	Lapatinib (EGFR inhibitor, at 2 µM) and YM155 (ABCB1 blocker, at 6.5 nM) treatment of tumor-bearing adult fish	Lapatinib is in clinical trials for the treatments of various types of cancers (see NCI clinical trial information).
Tao, T. et al., 2017 [50]	I-SceI meganuclease mediated transgenesis	*Tg(dβh:mCherry;dβh:DEF) and Tg*(*dβh:EGFP*;*dβh:MYCN*) transgenic fish lines	N/A	N/A
Zimmerman, M. W. et al., 2018 [51]	I-SceI meganuclease mediated transgenesis	*Tg*(*dβh:c-MYC*; *dβh:mCherry*) transgenic fish line	N/A	N/A
Shen, J. et al., 2018 [52]	Injection of tumor cells into the yolk sac of zebrafish embryos	Zebrafish embryos xenografted with SK-N-BE(2)-C human NB cell line	Crizotinib (ALK/MET inhibitor, at 8 μM) and 20a (histone deacetylase inhibitor, at 100 μM) treatment of embryos transplanted with SK-N-BE(2)-C human NB cells.	Crizotinib is in clinical trials for the treatments of various types of cancers, including high-risk NB (see NCI clinical trial information).
Aveic, S. et al., 2018 [53]	Injection of tumor cells into the duct of Cuvier of zebrafish embryos	*Tg(fli1:GFP)* zebrafish embryos transplanted with NB3 and SH-SY5Y NB cell lines	TP-0903 (multi-kinase inhibitor) treatment of embryos transplanted with NB3 and SH-SY5Y NB cell lines	TP-0903 is in a clinical trial for the treatment of FLT3 mutated acute myeloid leukemia (see NCI clinical trial information).
Seda, M. et al., 2019 [54]	Compound screen using *Tg(sox10:gfp)* transgenic larvae	N/A	Leflunomide was one of the top hits identified from a library of 640 compounds to regulate cartilage remodelling and NB cell viability.	Leflunomide is approved by FDA for the treatment of active rheumatoid arthritis. It is also in clinical trials for the treatments of various types of cancers (see NCI clinical trial information).
Koach, J. et al., 2019 [55]	Tol2 transposon- mediated transgenesis	*Tg(dβh:PA2G4)* transgenic fish line	WS6 (175.4 mg/kg, 5 μL) treatment of tumor-bearing fish	WS6 can also suppress tumor growth in the *TH*-*MYCN* mouse model and mice xenografted with human NB cell lines [55].
Tao, T. et al., 2020 [56]	I-SceI meganuclease mediated transgenesis	*Tg(dβh:EGFP;dβh:LIN28B_WT)* and *Tg(dβh:EGFP;dβh:LIN28B_MU)* transgenic fish lines	N/A	N/A
Shi, H. et al., 2020 [57]	CRISPR/Cas9-mediate gene knockout	*arid1aa* and *arid1ab* knockout fish lines	N/A	N/A
Dong, Z. et al., 2021 [58]	TALEN-mediate gene knockout	*gas7* knockout fish line	N/A	N/A
